# Survival and clinicopathological significance of PYCR1 expression in cancer: A meta-analysis

**DOI:** 10.3389/fonc.2022.985613

**Published:** 2022-09-02

**Authors:** Yue Li, Jiahuan Xu, Pengchen Bao, Zhijing Wei, Lei Pan, Jiawei Zhou, Wei Wang

**Affiliations:** ^1^ Department of Respiratory and Critical Care Medicine, The First Hospital of China Medical University, Shenyang, China; ^2^ China Medical University, Shenyang, China

**Keywords:** PYCR1, cancer, meta-analysis, prognosis, survival

## Abstract

**Background:**

Proline metabolism is closely related to the occurrence and development of cancer. Δ1-Pyrroline-5-carboxylate reductase (PYCR) is the last enzyme in proline biosynthesis. As one of the enzyme types, PYCR1 takes part in the whole process of the growth, invasion, and drug resistance of cancer cells. This study investigated PYCR1 expressions in cancers together with their relationship to clinical prognosis.

**Methods:**

A thorough database search was performed in PubMed, EMBASE, and Cochrane Library. RevMan5.3 software was used for the statistical analysis.

**Results:**

Eight articles were selected, and 728 cancer patients were enrolled. The cancer types include lung, stomach, pancreatic ductal adenocarcinoma, hepatocellular carcinoma, and renal cell carcinoma. The meta-analysis results showed that the expression of PYCR1 was higher in the clinical stage III–IV group than that in the clinical stage I–II group (OR = 1.67, 95%CI: 1.03–2.71), higher in the lymph node metastasis group than in the non-lymph node metastasis group (OR = 1.57, 95%CI: 1.06–2.33), and higher in the distant metastasis group than in the non-distant metastasis group (OR = 3.46, 95%CI: 1.64–7.29). However, there was no statistical difference in PYCR1 expression between different tumor sizes (OR = 1.50, 95%CI: 0.89–2.53) and degrees of differentiation (OR = 0.82, 95%CI: 0.54–1.24).

**Conclusion:**

PYCR1 had a high expression in various cancers and was associated with cancer volume and metastasis. The higher the PYCR1 expression was, the poorer the cancer prognosis was. The molecular events and biological processes mediated by PYCR1 might be the underlying mechanisms of metastasis.

## Introduction

Cancer is a global major public health issue seriously threatening human’s health and also a social heavy burden worldwide. According to the latest data from the World Health Organization’s Global Cancer Observatory, there were approximately 18.08 million new cancer cases worldwide in 2018 (1). Despite the rapid development in the etiology, diagnosis, and treatment of cancer, the prognosis of malignant tumors still remains not optimal. At present, the wide application of targeted therapy provides the treatment of cancer with a broad prospect. The key to targeted therapy is to find ideal molecular markers which can represent therapeutic and prognostic value and contribute to the patients’ risk stratification and optimal treatment selection.

Proline, a unique non-essential amino acid in humans, has been recognized as a structural disruptor and indicator of various pathological stresses during tumorigenesis (2–4). Proline metabolism features prominently in the unique metabolism of cancer cells (5). Proline and its associated metabolites and metabolic pathways are central to cancer growth and metastasis (6) and play an important role in poor prognosis. The broad effects of proline metabolism on the growth and survival of cancer cells suggest that proline-metabolizing enzymes can be a potential target for therapeutic intervention. Pyrroline-5-carboxylate reductase (PYCR), the final enzyme in proline biosynthesis, catalyzes the NAD(P)H-dependent reduction of Δ1 pyrroline-5-carboxylate methyl ester (P5C) to proline acid. The human genome encodes three isoforms of human PYCR: PYCR1, PYCR2, and PYCR3. PYCR1 is a mitochondrial inner membrane protein with 319 amino acids (UniProt P32322, chromosome 17q25.3, gene symbol PYCR1). It has been demonstrated to significantly affect cellular energy as well as physiological and pathological processes (7).

A study about the mRNA profiles of 19 types of cancers found that the expression of many metabolic genes was altered when compared with that in normal controls, and PYCR1 is one of the most frequently overexpressed metabolic genes (8). The high expression of PYCR1 is associated with poor prognosis in most cancer patients, suggesting that PYCR1 may be a potential target for cancer therapy. Until now, multiple studies have investigated the role of PYCR1 in various cancers, but most individual studies have their limitations, such as a small sample size or controversial results. Therefore, in order to explore the clinical prognostic value of PYCR1 in various cancers, we conducted a meta-analysis of clinical studies on PYCR1 and cancer, aiming to provide more reliable evidence for basic research and clinical work.

## Methods

### Search strategy

Two authors (YL and JX) independently searched the literatures by keywords (“Δ1-pyrroline-5-carboxylic acid reductase” OR “PYCR1”) AND (“cancer” OR “tumor”) and related synonym extensions from Cochrane Library, PubMed, CNKI, Tsinghua Tongfang, Wanfang Data, China Biomedical Literature Database, and China Academic Journal Full-text Database. The retrieval time of the literatures was from the establishment of the database to August 2022.

### Inclusion and exclusion criteria

The inclusion criteria were as follows: (1) published clinical studies providing original data on the relationship between PYCR1 expression and the pathology of cancer, (2) an immunoreactivity score based on staining intensity and proportion of stained cells was used for analysis, (3) all patients had complete clinical pathology results, and (4) odds ratio (OR) and 95%CI were provided by or could be calculated from the original literatures. For repeated publications out of the same population and with consistent data, the literature with the largest sample size was selected.

On the other hand, the exclusion criteria were as follows: (1) the positive criteria for PYCR1 detection were inconsistent, (2) unusable literatures such as duplicate reports, identical data, and poor quality, (3) reviews, comments, conference abstracts, and case reports, and (4) the subjects of the original research were not humans.

### Literature quality evaluation

Two authors (YL and JX) independently assessed the bias risk of the included studies, and by consensus, discrepancies were resolved together with the third author. The list includes 11 items. Each item was rated by “yes”, “no”, or “unclear”. One score was assigned to each item if the study satisfied the methodological criteria. For ratings of “no” or “unclear”, zero point was assigned. A score of 0 to 3 indicated poor quality, a score of 4 to 7 was of moderate quality, and a score of 8 to 11 meant high-quality research.

### Data extraction

Two researchers (YL and JX) independently extracted data from the selected literatures and carried out cross-checking. The extracted information included the following: first author, publication date, country, type of research design, basic information of the research subjects, and the amount of samples. For differences of the extracted data, the two researchers reached an agreement through a discussion, and if a dispute remained, a third researcher would come forward to adjudicate.

### Statistical method

Meta-analysis was performed using RevMan5.3. All ORs and 95%CIs were combined to assess the impact of high PYCR1 expression on prognosis. Heterogeneity analysis was performed by *X* ^2^ test, and *I* ^2^ was used to quantitatively evaluate the magnitude of heterogeneity. If *I* ^2^ ≤50%, a fixed effect model was used; if *I* ^2^ >50%, the source of heterogeneity was analyzed, and it was considered whether to use a random effect model to combine analysis or not, and sensitivity analysis or subgroup analysis was performed. The overall test effect was based on *P <*0.05, the specific data was combined and embodied by a forest plot, and a funnel plot was used to evaluate publication bias.

## Results

### Literature screening results

**Figure 1:**
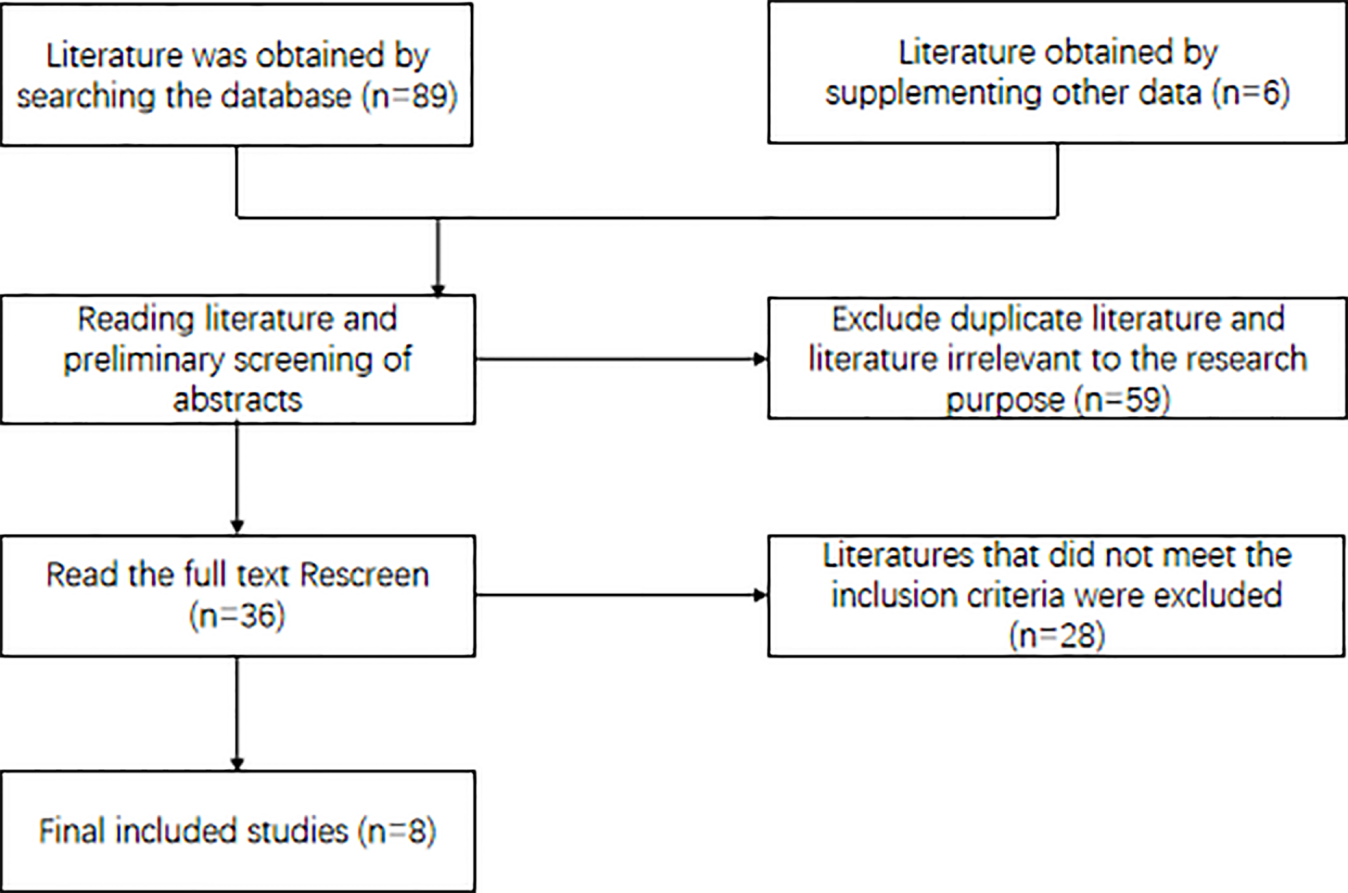
Literature retrieval process and results.

According to the established search strategy, 89 literatures were initially screened. After exclusion according to the literature screening flow chart (**Figure 1**), eight clinical research literatures were finally included, and the studied population consisted of 728 cases (9–16). The literature was of high quality and without significant publication bias. **Table 1** Basic characteristics of included studies (**Table 1** shows).

**Table 1:** Basic characteristics of the included studies.

References	Years	Type of cancer	Case (*N*)	Analytical metrics
**Feng**, **C**	2017	Non-small cell lung cancer	62	Degree of differentiation, tumor size, clinical stage, and lymph node metastasis
**Gao**, **YW**	2020	Non-small cell lung cancer	60	Clinical stage and lymph node metastasis
**Wang**, **DC**	2019	Non-small cell lung cancer	145	Degree of differentiation, clinical stage, and lymph node metastasis
**Xiao**, **SY**	2020	Stomach cancer	90	Degree of differentiation, tumor size, clinical stage, lymph node metastasis, and distant metastasis
**Xu**, **YZ**	2021	Hepatocellular carcinoma	106	Tumor size, clinical stage, and distant metastasis
**Fu**, **WJ**	2019	Renal cell carcinoma	96	Degree of differentiation, tumor size, clinical stage, and distant metastasis
**Wang**, **HY**	2022	Pancreatic ductal adenocarcinoma	89	Degree of differentiation, tumor size, clinical stage, and lymph node metastasis
**Cheng**, **QH**	2021	Hepatocellular carcinoma	80	Tumor size and clinical stage

### Quality evaluation results

The six included articles covered two 4 points, three 5 points, and three 8 points.

### Meta-analysis results

**Figure 2:**
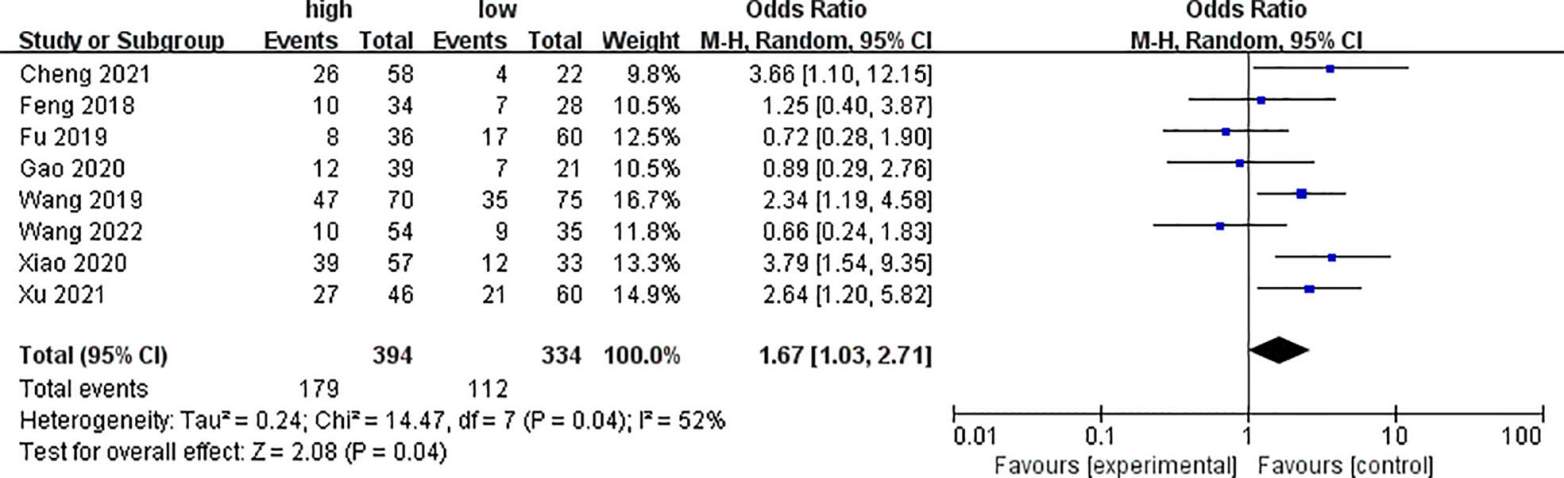
Forest plot of meta-analysis of the relationship between PYCR1 expression and the clinical stage of cancer.

Eight of the included literatures analyzed clinical stages, as shown in **Figure 2**. The analysis results showed that the expression of PYCR1 significantly increased in the clinical stage III–IV group (OR = 1.67, 95%CI: 1.03–2.71).

**Figure 3:**
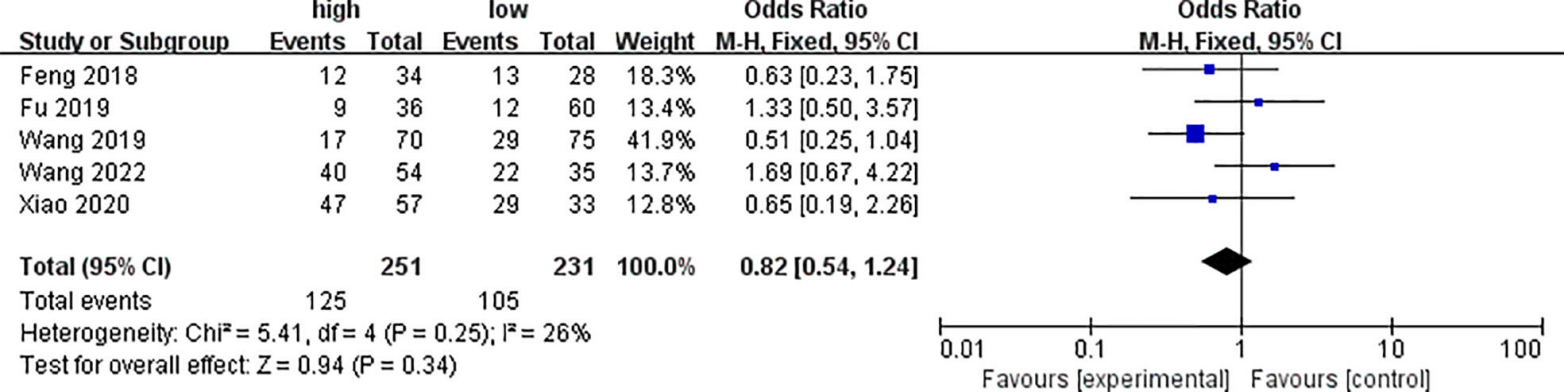
Forest plot of meta-analysis of the relationship between PYCR1 expression and cancer differentiation.

Five of the included literatures analyzed the differences of PYCR1 expression between different degrees of differentiation, as shown in **Figure 3**. The results showed that there was no significant correlation between cancers of different degrees of differentiation and PYCR1 expression (OR = 0.82, 95%CI: 0.54–1.24).

**Figure 4:**
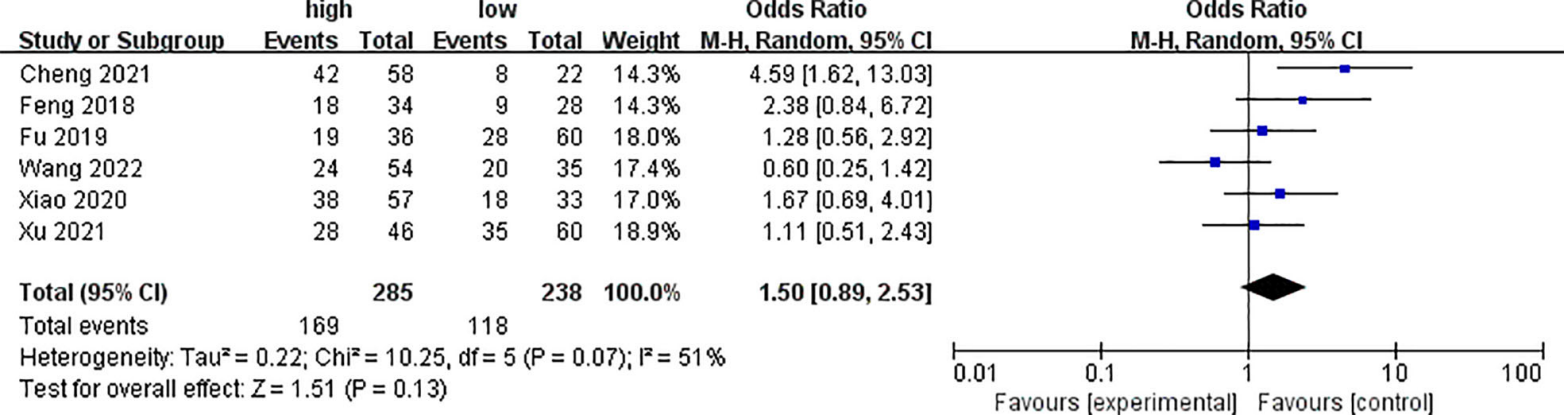
Forest plot of meta-analysis of the relationship between PYCR1 expression and cancer size.

Six of the included studies analyzed the differences of PYCR1 expression between different cancer sizes, as shown in **Figure 4**. The results showed that there was no significant correlation between cancers of different sizes and PYCR1 expression (OR = 1.50, 95%CI: 0.89–2.53).

**Figure 5:**
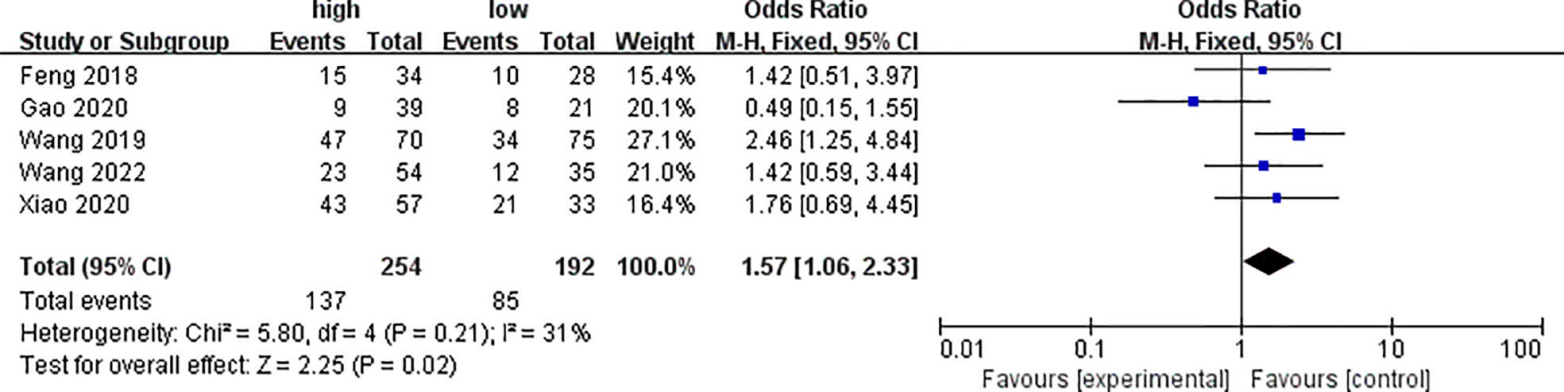
Forest plot of meta-analysis of the relationship between PYCR1 expression and cancer lymph node metastasis.

Five of the included studies analyzed the differences of PYCR1 expression among different lymph node metastasis statuses, as shown in **Figure 5**. The results showed that the expression of PYCR1 in the lymph node metastasis group significantly increased (OR = 1.57, 95%CI: 1.06–2.33).

**Figure 6:**
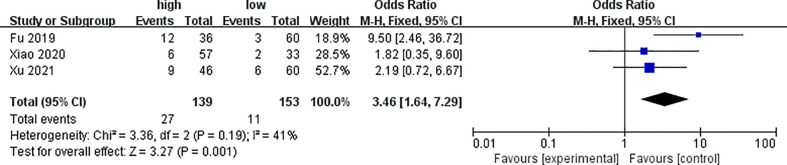
Forest plot of meta-analysis of the relationship between PYCR1 expression and distant metastasis of cancer.

Three of the included studies analyzed the differences of PYCR1 expression between different distant metastasis states, as shown in **Figure 6**. The analysis results showed that the expression of PYCR1 in the distant metastasis group significantly increased (OR = 3.46, 95%CI: 1.64–7.29).

### Publication bias

**Figure 7:**
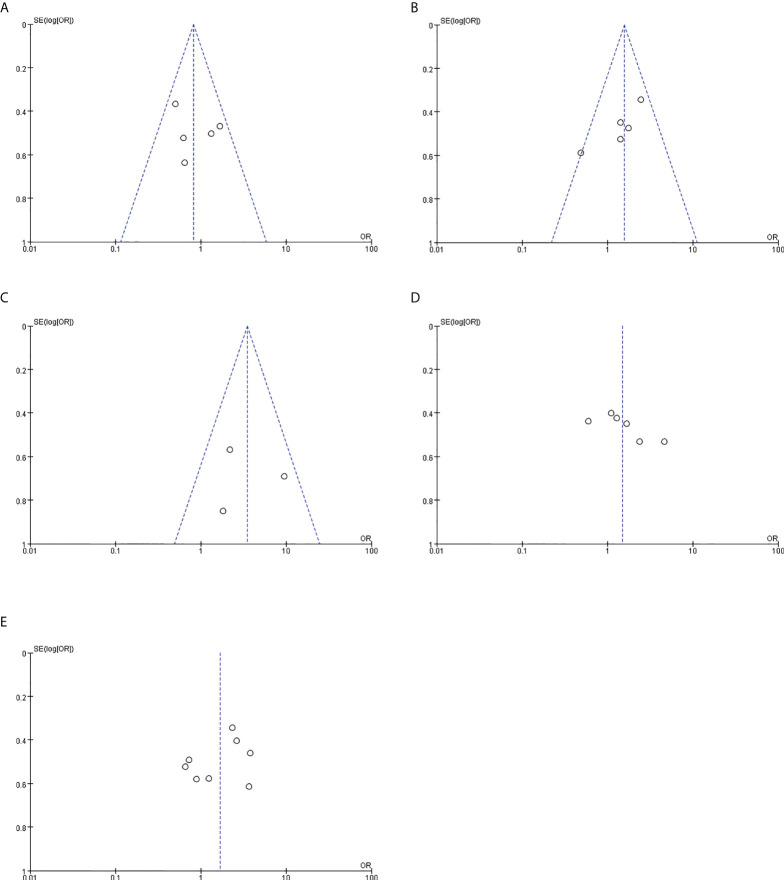
Publication bias. **(A)** Relationship between PYCR1 expression and cancer differentiation. **(B)** Relationship between PYCR1 expression and cancer lymph node metastasis. **(C)** Relationship between PYCR1 expression and distant metastasis of cancer. **(D)** Relationship between PYCR1 expression and cancer size. **(E)** PYCR1 expression and clinical stage of cancer relation.

Funnel plots were drawn for the clinical studies included in this study (**Figure 7**). Each study was distributed within the funnel range. However, the sample size of this study was small, and less than 10 clinical studies were included in this meta-analysis, so the effect of the funnel plot was not sufficient for evaluating publication bias.

## Discussion

As an enzyme in proline metabolism, PYCR1 has a trend of increased expression in various cancers, which has been recognized by more and more researchers. It has been reported that, compared with normal tissues, PYCR1 gene expression was consistently higher in cancer tissues (17), and knockdown of PYCR1 impaired cancer cell proliferation (18–21). PYCR1 gene expression could predict poor cancer characteristics and patient outcomes (22, 23). These have been observed in many different types of cancer. However, no meta-analysis has been performed to evaluate the prognostic value of a high expression of PYCR1 in cancer patients up to now. This is the first comprehensive meta-analysis about the influence of increased PYCR1 expression on the survival and clinicopathological characteristics of cancer. In this study, we enrolled five different cancer types (non-small cell lung cancer, gastric cancer, hepatocellular carcinoma, pancreatic ductal adenocarcinoma, and renal cell carcinoma) and performed a meta-analysis of data from 728 patients to assess the significance of PYCR1 expression in cancer prognosis. We found that PYCR1 overexpression was significantly associated with higher cancer TNM stage, lymph node metastasis, and distant metastasis stage but not with cancer differentiation and cancer size.

In 2020, Anjana *et al.* found that PYCR1 protein levels were significantly higher than normal levels in breast cancer samples before treatment, decreased after treatment, but still significantly higher than normal levels, and high levels of PYCR1 in residual tumors were associated with short overall survival (24). Our findings confirmed that PYCR1 expression was strongly correlated with clinical features (tumor TNM stage, lymph node metastasis, and distant metastasis stage). Zhuang *et al.* used SMMC-7721 cells to perform xenograft experiments in nude mice; a significantly increased cancer volume was observed in the control group treated with shCtrl, while cancer was not observed in the experimental group treated with shPYCR1. These results suggested that PYCR1 interference could inhibit cancer growth *in vivo* (25). Second, some studies *in vitro* focused on the relationship between PYCR1 and cancer proliferation and migration. Under normal circumstances, the proliferation of cancer cells is prone to hypoxia in the local microenvironment, and hypoxia can cause tumor proliferation to stop, but Westbrook *et al.* found that cancer cells can support their continued proliferation under hypoxic conditions through increased PYCR1 activity during hypoxia (26). Zeng *et al.* knocked down PYCR1 in prostate cancer cells by lentivirus-mediated gene delivery and performed cell cycle and apoptosis detection assays. They found that a low expression of PYCR1 resulted in the inhibition of cell proliferation and colony formation, cell cycle arrest, and apoptosis in prostate cancer cells (27). Guo et al. carried out siRNA transfection of PYCR1 to knock down the expression of PYCR1 in human hepatocellular carcinoma cell lines and performed CCK-8, clone formation assay, flow cytometry, and western blotting to detect key proteins related to apoptosis. Their results showed the decreased number of cell clones and the increased percentage of apoptotic cells in the siRNA transfection group when compared to the control group. Furthermore, knockdown of PYCR1 could downregulate Bcl-2 expression and upregulate Bax and Caspase3 expression, indicating that PYCR1 inhibits apoptosis (28). Third, PYCR1 could also induce cancer progression by enhancing cell migration and invasion ability. Increased production of collagen-rich extracellular matrix is a hallmark of cancer-associated fibroblasts (CAFs) and a major driver of cancer aggressiveness. Kay *et al.* confirmed PYCR1 upregulation in all CAF lines by western blot analysis, and by targeting PYCR1 in CAFs in a breast cancer co-transplantation model, the size and weight of tumors in the PYCR1-expressing group were reduced, and the surrounding fibrillar collagen was significantly reduced, compared with the control group, which showed that reducing the PYCR1 levels in CAFs reduced tumor collagen production, tumor growth, and metastatic spread *in vivo* and cancer cell proliferation *in vitro* (29 **).** Du *et al.* found that PYCR1 knockdown significantly reduced Akt phosphorylation and inhibited the activation of Wnt/β-catenin signaling, thereby significantly inhibiting bladder tumor formation. However, when AKT was inhibited, the progression of tumor was reversed by over-expressed PYCR1 (30). How dose PYCR1 carry out its role in tumor progression? Yan *et al.* used the ChIPBase database to search and predict the target genes of PYCR1 and found that STAT3, as a signal transducer and activator of transcription, participated in various signaling pathways related to cancer progression together with PYCR1. To further explore the mechanisms involved in PYCR1-regulated proliferation, drug resistance, and EMT when PYCR1 was knocked down in colorectal cancer cells, the researchers observed that the STAT3-mediated p38 MAPK and NF-κB signaling pathways were inhibited, while a simultaneous overexpression of STAT3 could partially reverse the effects of PYCR1 on colorectal cancer cell proliferation, drug resistance, and EMT (31). These suggested that PYCR1 played a key role in enhancing cell migration and invasion through the above-mentioned mechanisms. Fourth, cancer recurrence caused by chemotherapy resistance is a major clinical challenge for advanced cancer. Current studies have shown that PYCR1 is involved in the process of cancer resistance. Doxorubicin inhibits cancer proliferation by interfering with cancer cell DNA synthesis and is the most common therapeutic agent for breast cancer. In their *in vitro* studies, Ding *et al.* found that inhibition of PYCR1 significantly enhanced the effect of doxorubicin cytotoxicity in breast cancer cell lines MCF-7 (ER positive) and MDA-MB-231 (ER negative). It was also observed in population studies that chemotherapy significantly improved the survival rate of patients with low PYCR1 expression at the early stage of breast cancer, which confirmed that the lack of PYCR1 might enhance the chemosensitivity of doxorubicin to breast cancer (32). When treating lung adenocarcinoma cells with cisplatin in PYCR1-silenced/vehicle control/blank control groups, cisplatin stimulation significantly increased cell proliferation in PYCR1-silenced group when compared to the vehicle control. The results indicated that PYCR1 silencing could increase cisplatin sensitivity to lung adenocarcinoma cells (33). Fifth, the current study found that inhibition of PYCR1 can also enhance the effect of other anti-tumor drugs. SK (shikonin) is a small-molecule naphthoquinone compound that has been shown to have various effects, such as anti-inflammatory, antiviral, antitumor, and immunomodulatory properties (34, 35). Zhang *et al.* found that SK and its derivative shikonin minimized the expression of PYCR1 protein and mRNA in hepatoma cells. When the expression of PYCR1 was downregulated, it enhanced the inhibitory effect of SK on hepatoma cells. The combined treatment of PYCR1 knockout and SK significantly inhibited cell proliferation, cell migration, and cell invasion compared to SK treatment alone (36). Oudaert *et al.* treated multiple myeloma cells with the PYCR1 inhibitor pargyline in combination with bortezomib and found increased bortezomib-mediated apoptosis. Combination treatment with pargyline and bortezomib reduced the viability of CD138+ MM cells compared to a single agent, and tumor burden was assessed by eGFP positivity on flow cytometry. Combination therapy was found to reduce tumor burden in a syngeneic immunocompetent 5TGM1 mouse model (37).

There were some potential limitations in this study. Due to the frontier position at present about the clinical research on the correlation between PYCR1 expression level and cancer prognosis, we only observed the prognostic value in five types of cancers (non-small cell lung cancer, gastric cancer, pancreatic ductal adenocarcinoma, hepatocellular carcinoma, and renal cell carcinoma), while the value in other types of cancers was still unclear yet. However, animal experiments indicated that there were certain correlations in prostate cancer, colorectal cancer, and melanoma, that is to say, PYCR1 knockdown inhibited cancer progression. These suggested that PYCR1 expression did reflect the prognosis of many cancers. In addition, there is little data about the change of PYCR1 expression in cancer management. Further studies need be designed to test it.

In conclusion, overexpression of PYCR1 was a potential risk for poor prognosis in various cancers, and PYCR1 might serve as a potential cancer therapeutic target. Our findings provided clues for understanding the clinicopathological significance of PYCR1 expression in cancer and are of great value for guiding new targets for cancer therapy. It has been reported that the discovery of PYCR1 inhibitors is in the initial stage, and some studies have identified pargyline as an inhibitor of PYCR1 (38). The main challenge in the future is to determine the tractability of PYCR1 as a drug target.

## Data availability statement

The original contributions presented in the study are included in the article/supplementary material. Further inquiries can be directed to the corresponding author.

## Author contributions

YL contributed to data acquisition and drafted the manuscript. JX and LP contributed to the literature review, manuscript editing, and subsequent minor revision. PB, ZW, and JZ were involved in editing the manuscript. WW contributed to the study design and revision of the manuscript. All authors contributed to the article and approved the submitted version.

## Funding

This work was supported by the Distinguished Professor of Liaoning Province [Liao Jiao Fa (2015) no. 153].

## Acknowledgments

We are grateful to all the authors for their individual contributions to the present study.

## Conflict of interest

The authors declare that the research was conducted in the absence of any commercial or financial relationships that could be construed as a potential conflict of interest.

## Publisher’s note

All claims expressed in this article are solely those of the authors and do not necessarily represent those of their affiliated organizations, or those of the publisher, the editors and the reviewers. Any product that may be evaluated in this article, or claim that may be made by its manufacturer, is not guaranteed or endorsed by the publisher.
